# Supplementary material for: Fat Embolism Syndrome in Sickle Cell Disease
Source: J Clin Med. 2020 Nov 8;9(11):3601. doi: 10.3390/jcm9113601 (PMC7695297; doi:10.3390/jcm9113601)
Supplement: Supplementary file 1 [file jcm-09-03601-s001.pdf]

Algorithm for early recognition and management of fat embolism syndrome in adult patients with Sickle Cell Disease

Patients at risk for developing FES:
Previous mild disease, infrequent hospital attendance usually HbSC or Hb $S\beta^{+}$

Check: FBC, reticulocytes, renal function, liver function, **ferritin, LDH**, CRP, request blood film and send group and save

Presenting with pain only

<u>Monitor very closely for</u> → (Presentation with any of)

Consider pre-emptive exchange transfusion if

- Sever pain AND
- Ferritin > 20 x baseline AND ONE OF
    - LDH > 20 x baseline
    - Thrombocytopenia or platelets < 50% from baseline
    - Fever

Discuss <u>immediately</u> with haematology

Presentation with any of

- Respiratory failure
- Neurology (altered mental status, focal neurology, coma)
- Other organ dysfunction eg liver, renal, cardiac
- Suggestive labs

↓

Possible FES

↓

Immediate actions

- Inform haematology /ITU
- Haematology to decide emergency exchange transfusion

Emergency red cell exchange transfusion (RCE) <u>as soon as FES is suspected</u>

- if pre-transfusion sickle level (HbS or HbSC) unknown consider it 100%
- Target post transfusion sickle level < 20% (Hb S or HbS&C for HbSC patients)

↓

Therapeutic plasma exchange (TPE~~X~~)

- TPE~~X~~ to follow RCE unless complete clinical recovery and improvement in ferritin, LDH and platelets
- Exchange 1 plasma volume/day for 5 days using Octaplas as replacement fluid (alternatively FFP)
- Monitor clinically, ferritin, LDH and platelets
- May need to continue beyond 5 days
